# Figures in clinical trial reports: current practice & scope for improvement

**DOI:** 10.1186/1745-6215-8-36

**Published:** 2007-11-19

**Authors:** Stuart J Pocock, Thomas G Travison, Lisa M Wruck

**Affiliations:** 1 London School of Hygiene and Tropical Medicine, London, UK; 2 New England Research Institutes, Watertown, MA, USA; 3 Now at Rho Inc., Chapel Hill, NC, USA

## Abstract

**Background:**

Most clinical trial publications include figures, but there is little guidance on what results should be displayed as figures and how.

**Purpose:**

To evaluate the current use of figures in Trial reports, and to make constructive suggestions for future practice.

**Methods:**

We surveyed all 77 reports of randomised controlled trials in five general medical journals during November 2006 to January 2007. The numbers and types of figures were determined, and then each Figure was assessed for its style, content, clarity and suitability. As a consequence, guidelines are developed for presenting figures, both in general and for each specific common type of Figure.

**Results:**

Most trial reports contained one to three figures, mean 2.3 per article. The four main types were flow diagram, Kaplan Meier plot, Forest plot (for subgroup analyses) and repeated measures over time: these accounted for 92% of all figures published. For each type of figure there is a considerable diversity of practice in both style and content which we illustrate with selected examples of both good and bad practice. Some pointers on what to do, and what to avoid, are derived from our critical evaluation of these articles' use of figures.

**Conclusion:**

There is considerable scope for authors to improve their use of figures in clinical trial reports, as regards which figures to choose, their style of presentation and labelling, and their specific content. Particular improvements are needed for the four main types of figures commonly used.

## Introduction

Much has been written about how to visually display quantitative information, [1-4] and some attention has been paid to the specific constraints of including figures in Medical journal articles [5-9]. In this article we focus on the use of figures in reports of randomised clinical trials, for which there is little specific guidance available at present[10].

In order to understand current practice, we undertook a survey of recent publications of randomized clinical trial in major general medical journals. This provides objective evidence as to the extent of use of figures in trial reports, including which types of figure are chosen by authors. This survey then facilitates constructive critical appraisal of both the good features and the limitations of how authors and journals are utilizing figures for visual display of results.

In formulating our recommendations for improved use of figures in future trial reports, we give particular attention to each main type of figure used and draw on published examples to illustrate the specific features of interest.

## Methods

We identified all 77 reports of randomised clinical trials published in November 2006 to January 2007 in the following five major general medical journals: Archives of Internal Medicine, British Medical Journal (BMJ), Journal of American Medical Association (JAMA), Lancet and New England Journal of Medicine (NEJM). BMJ publishes a shorter version of each article in print, and a larger version on-line. We have studied the longer versions.

For each article we first noted the number and types of figure included, restricting attention to figures displaying data. There were nine other Figures, mostly schema of trial protocols. From past guidance in the literature and our own previous surveys of trial reports in 1999 [11,12] and 2005 [13] we formulated a prior list of issues that we thought were pertinent to the style and content of both figures in general and the specific common types of figures used in trial reports. With this list in mind, we carefully inspected every figure included in our survey regarding the appropriateness of its presentation and content. This exercise led us to refine our list (presented as recommendation at end of the Discussion) as to what constitutes good and bad practice in the use of figures, and to select specific examples to add practicality to our illustrative points.

## Results

Table 1 displays the main facts from our survey. In this three month period, the New England Journal published more trial reports than each of the other four journals. Most articles contained one, two or three figures and there were 175 figures in 77 articles, mean 2.3 figures per article.

**Table 1:** Figures in Reports of Clinical Trials in Five Medical Journals during November 2006 to January 2007.

**Journal**	**No. of articles (total 77)**
Annals of Internal Medicine	7
British Medical Journal	16
Journal of American Medical Association	14
Lancet	12
New England Journal of Medicine	28
**No. of Figures in each article**	
None	1
One	23
Two	22
Three	19
Four	11
Seven	1
Types of Figure in each article*	
Flow Diagram	66
Kaplan Meier plot	32
Forest plot	21
Repeated measures	20
Bar chart	7
Individual patient data	3
Box plot	2
Cumulative distribution	1

*Some articles had one type of Figure several times. In this Table each type of Figure is only counted once per article.

The most common types of figure were:

**Flow diagram **(66 articles) describing the flow of participants through the various stages of the trial.

**Kaplan Meier plot **(32 articles) comparing treatments for time-to-event (survival) outcomes.

**Forest plot **(21 articles) displaying several estimates of treatment effect, usually by subgroups of patients, but occasionally by other comparative features.

**Repeated measures plot **(20 articles) displaying mean outcomes at baseline and several follow-up times by treatment group.

These four types of plot accounted for 92% of figures in our survey. The remainder comprised **bar chart **(7 articles), **individual patient data display **(3 articles), **box plot **(2 articles), **cumulative distributions **(1 article).

We now turn attention to the style and content of specific types of figure. From the survey, we have chosen four examples of each main type of Figure, plus a few other examples (20 examples in all) to illustrate the main features to consider, include, and sometimes avoid, in one's use of figures.

The flow diagram is an integral part of the CONSORT guidelines [14,15], adopted by most major journals. Hence it is meant to be a mandatory requirement for publication in all journals we surveyed, except NEJM which had flow diagrams for half its clinical trial articles. Its aim is to display the flow of participants through each stage, specifically for each randomized group reporting the numbers randomly assigned, receiving intended treatment, completing study protocol, and analysed for the primary outcome.

**Figure 1:**
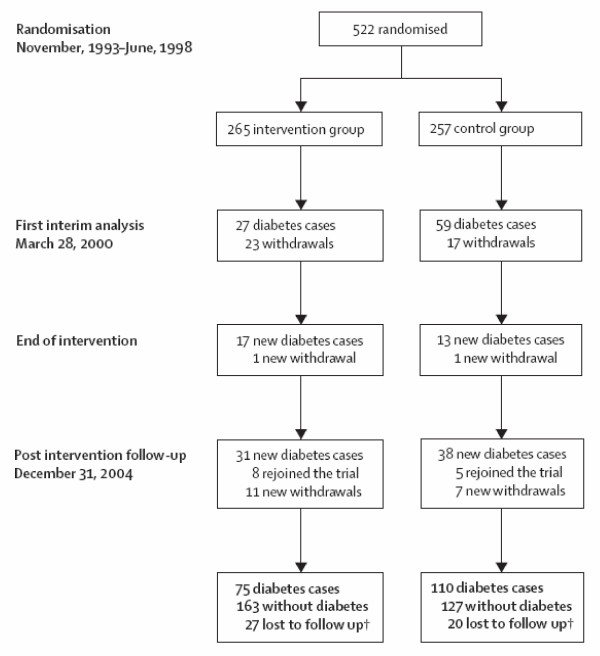
**Trial profile**. *After the decision to end the intervention period, the intervention was continued until each participant's next scheduled yearly clinic visit. End date thus varied from March, 2000, to Dec, 2001. † Participants who were lost to follow-up were treated as censored observations in the analyses. *(Lindstrom et al.  Lancet Nov 11, 2006 p1674)*

Figure 1 is a straightforward example. One limitation is that it does not reveal who the participants are and what type of intervention was received. This could have been achieved by line one starting "522 randomized participants with impaired glucose tolerance", and line two inserting "life-style" before "intervention". Information on loss-to-follow up is important. This figure helpfully gives the numbers experiencing the primary outcome, so that the readers see upfront that few diabetic cases occurred in the intervention group.

**Figure 2:**
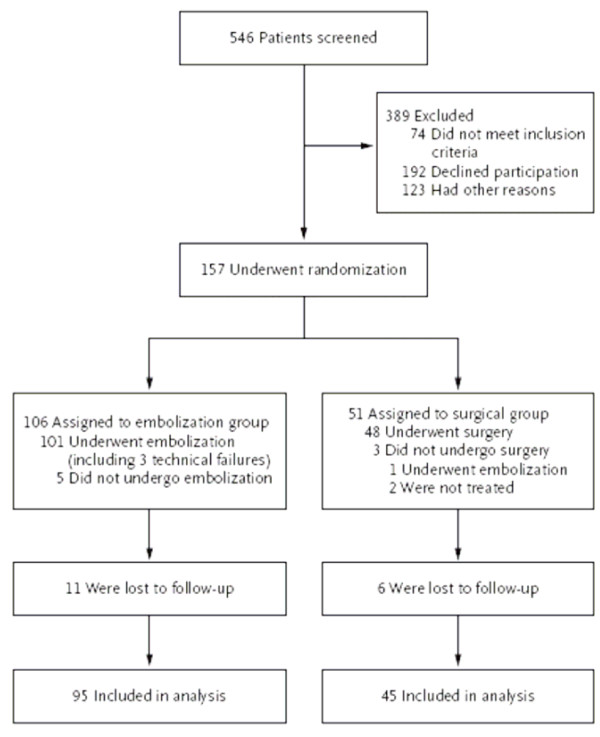
Enrollment and Outcomes. (*REST Investigators. NEJM Jan 25, 2007 p362*)

Figure 2 includes some useful extra features: the numbers of patients screened for potential inclusion, the reasons for exclusions from randomization, and the numbers who did not receive their intended treatment.

**Figure 3:**
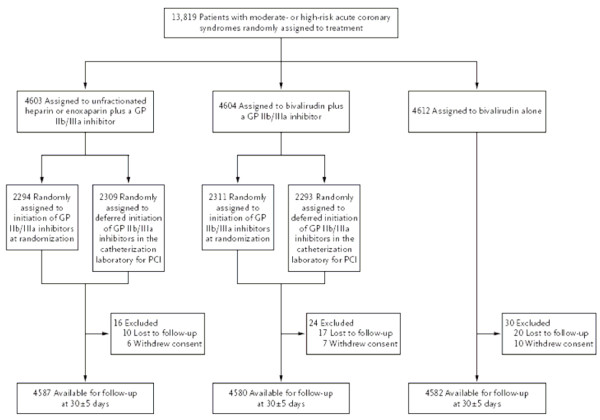
**Enrollment, Randomization, and Follow-up of the Patients in the Trial**. GP denotes glycoprotein. *(Stone et al.  NEJM Nov 23, 2006 p2205)*

In trials with a more complex design, the flow diagram is especially useful. For instance, Figure 3 illustrates a partial two way factorial design, in which the second randomization concerned the timing of one treatment. To clarify the full extent of randomization, row two could have inserted "randomly" before "assigned" each time.

**Figure 4:**
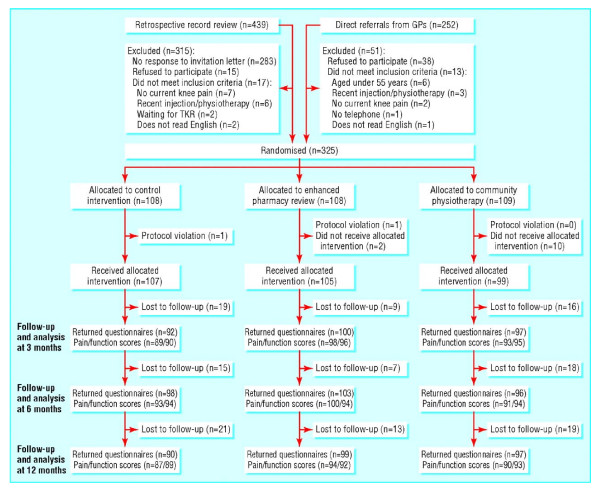
**Trial profile**. GP = general practitioner; TKR = total knee replacement. *(Hay et al.  BMJ online Nov 11, 2006 p3)*

Figure 4 illustrates one stylistic problem in flow diagrams, which is that words can get repeated many times in routine display with more than two treatments. Thus if this figure had been displayed as a table instead, with appropriate row and column headings, the numbers of words would have been reduced by more than half, with no loss of information.

The Kaplan Meier plot is the routine method of displaying time-to-event (survival) data by treatment group [12]. The event may be death, a non-fatal event (e.g. disease recurrence), a composite outcome (e.g. time to death, myocardial infarction or stroke whichever occurs first) or occasionally a good outcome (e.g. time to recovery).

**Figure 5:**
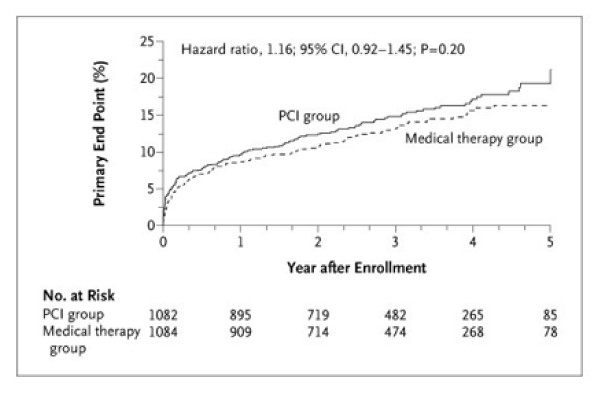
**Kaplan-Meier Curves for the Primary End Point, According to the Intention-to-Treat Analysis**. The primary end point was the first centrally adjudicated occurrence of death from any cause, nonfatal reinfarction, or NYHA class IV heart failure requiring hospitalization or a stay in a short-stay unit. Kaplan-Meier estimates of the cumulative event rates in the PCI group and the medical therapy group, respectively, were 14.8% and 13.1% at 3 years, 17.2% and 15.6% at 4 years, and 21.2% and 16.4% at 5 years. The cumulative yearly adjusted hazard ratios for PCI versus medical therapy for years 1 through 5 were 1.13, 1.18, 1.14, 1.13, and 1.16, respectively. The P value was calculated with the use of the log-rank test. *(Hochman et al.  NEJM Dec 7, 2006 p2403)*

Figure 5 illustrates many of the essential features. The two treatment groups are clearly identifiable, and the axes clearly labelled. This concerns the study's primary endpoint, which is defined in the figure's footnote. The graph is plotted going up, i.e. cumulative % incidence, with an appropriate maximum (25%) on the vertical scale which allows the detail to be seen. Underneath the horizontal axis the numbers at risk (i.e. still in follow-up and without the primary endpoint as yet) are shown at convenient (one year) intervals. In this case, the numbers in follow-up get rapidly smaller in the later years. Though not stated, it looks appears that median follow-up was around three years, so it might have been better not to extend the graph out to five years. The eye is naturally drawn to the right hand end of the graph where the estimated percentages become increasingly prone to random error.

A superficial glance at Figure 5 takes in the fact that the PCI group has a slightly higher % endpoints at all times, but this can be readily attributable to chance. Hence, it is good practice to include the hazard ratio, its 95% CI and the logrank P-value on the figure to clarify the (lack of) evidence concerning a treatment difference. Figure 5's footnote includes yet more details on treatment comparisons by year, which is perhaps more than is usually warranted.

**Figure 6:**
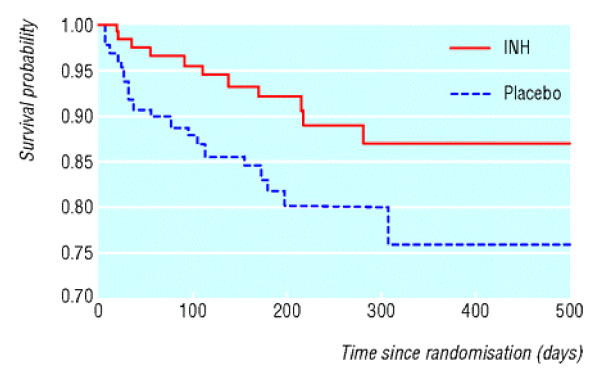
Survival in children on isoniazid (INH) or placebo. *(Zar et al.  BMJ online Jan 20, 2007 p4)*

By contrast, Figure 6 has several problems. The vertical axis is for proportion surviving rather than dead, and has a cut-off at 0.70. This tends to deceptively exaggerate any treatment differences. This is enhanced by the lack of information on numbers at risk over time (i.e. how many were censored before 500 days?) and the lack of any estimates, CIs or P-values on the graph. This is all clarified in the article: 11/132 versus 21/131 deaths, hazard ratio 0.46, 95% CI 0.22 to 0.95, logrank P = 0.015.

**Figure 7:**
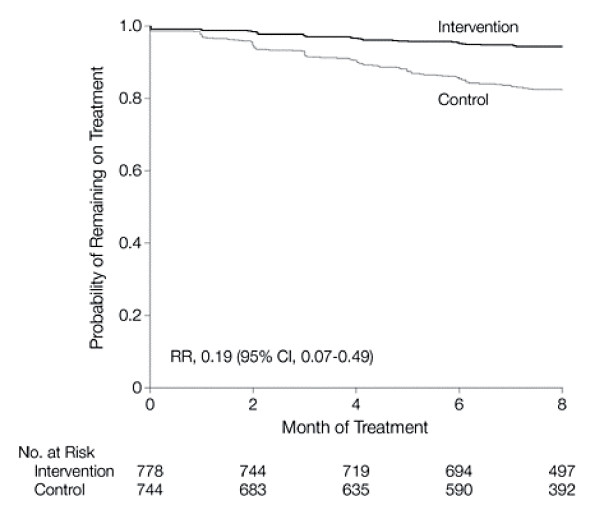
**Kaplan-Meier Curve of the Time to Defaulting From Treatment by Study Group**. RR indicates risk ratio; CI, confidence interval. *(Thiam et al.  JAMA Jan 24, 2007 p384)*

Figure 7 is an example of a Kaplan-Meier plot going down covering the whole vertical scale from probability 1 to probability 0. Since the event (defaulting from treatment) has a low occurrence, much of the graph is empty space. Perhaps such a plot going down is best kept for trials with high failure rates e.g. the low survival rates in studies of advanced cancer.

**Figure 8:**
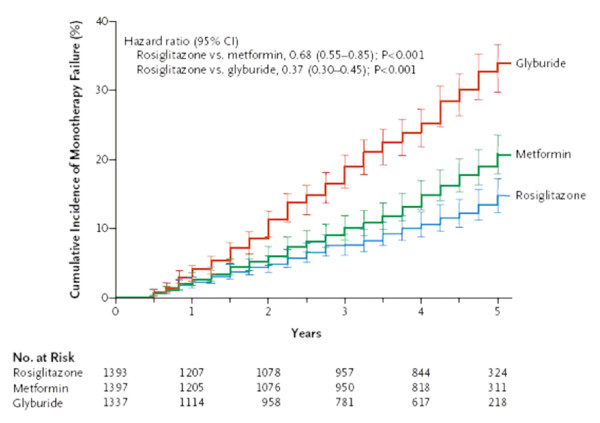
**Kaplan-Meier Estimates of the Cumulative Incidence of Monotherapy Failure at 5 Years**. Treatment was considered to have failed if a patient had a confirmed or adjudicated level of fasting plasma glucose of more than 180 mg per deciliter. Risk reduction is listed for comparisons of pairwise groups from a baseline covariate-adjusted Cox proportional-hazards model. Gray's estimates of cumulative incidence adjusted for all deaths were smaller than Kaplan-Meier estimates of treatment failure: 10% in the rosiglitazone group, 15% in the metformin group, and 25% in the glyburide group. I bars indicate 95% CIs. *(Kahn et al.  NEJM Dec 7, 2006 p2433)*

One problem with most Kaplan Meier plots is the lack of any display of statistical uncertainty, which may lead readers to over-interpret any observed treatment difference. Figure 8 is a relatively rare example where the plot includes 95% CIs for the estimates over time. The end result in this case is a bit too cluttered, so it might have been better if these had been included at each year rather than every three months. Also, standard error bars might be preferable as they are half the width of 95% CIs. Regardless of the method, displaying such uncertainty is to be encouraged. Note the stepped pattern to the plots in figure 8: this is appropriate because the outcome, monotherapy failure, was observed at each three-monthly visit rather than in continuous time.

**Figure 9:**
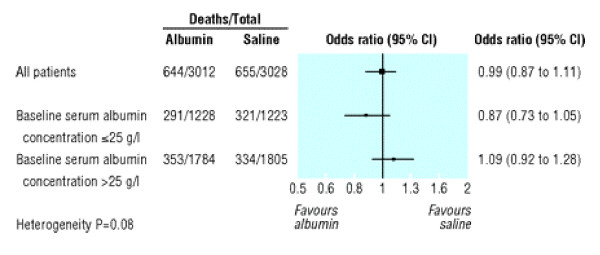
Unadjusted odds ratio (95% confidence interval) of death in all patients and in subgroups with baseline serum albumin concentration of 25 g/l or less and of more than 25 g/l. (Heterogeneity of treatment effect in subgroups with baseline serum albumin concentration ≤25 g/l *v *>25 g/l, P = 0.08). *(SAFE Study.  BMJ online Nov 18, 2006 p4)*

A Forest plot is a method of displaying the extent to which the estimated treatment effect differs across various subgroups of patient [16,17]. Figure 9 is a relatively simple example to explain. The estimate of treatment effect in this instance is the odds ratio of death for albumin compared to saline. The two subgroups are patients with baseline albumin below or above 25 g/l and for each the odds ratio and its 95% CI are plotted. Labels helpfully indicate that to the left of the vertical line at odds ratio equal to one favours albumin while to the right favours saline. Both CIs include one which indicates non-significance at the 5% level. However it is more meaningful to note that the two CIs overlap somewhat, suggesting there is insufficient evidence to claim an interaction between treatment and baseline albumin. This is made clear by the heterogeneity test (sometimes called interaction test) P = 0.08.

Note the horizontal axis is on a log scale i.e. the distance from 0.5 to 1 is the same as the distance from 1 to 2. This makes sense in that a halving and a doubling of odds are of equal magnitude. This use of log scale also makes all CIs symmetric about the estimated effects. The plot usefully gives the overall estimated odds ratio for all patients, and its CI. Figure 9 also gives in tabular form i) the number of deaths and patients by treatment overall and by subgroup and ii) the consequent odds ratios and CIs that are already plotted. This duplication of information is useful or repetitious, depending on the tastes of authors and editors.

**Figure 10:**
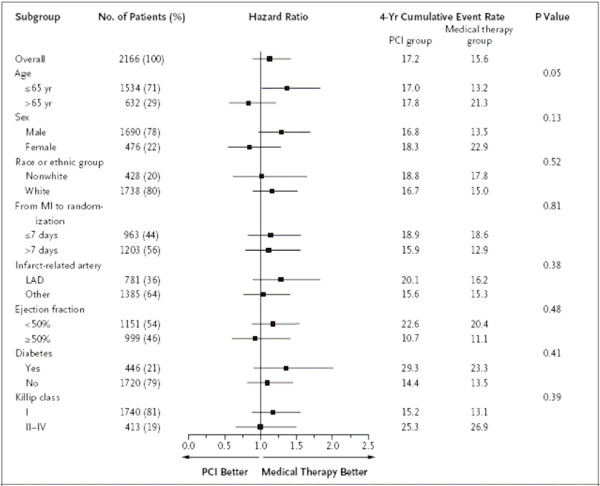
**Subgroup Analysis**. Hazard ratios (black squares), 95% CIs (horizontal lines), P values for the interaction between the treatment effect and any subgroup variable, and cumulative estimated 4-year event rates for the primary outcome (death from any cause, nonfatal reinfarction, or NYHA class IV heart failure requiring hospitalization or a stay in a short-stay unit) for PCI versus medical therapy for the specified subgroups are shown. Age, sex, race or ethnic group, the location of the infarct-related artery, the ejection fraction, and the time from the index myocardial infarction (MI) to randomization were prespecified. Race was self-reported. Diabetes and the highest Killip class during the index MI were not prespecified for the subgroup analysis. Originally, the cutoff point for age was 70 years, but early during the trial monitoring and before any analyses were performed, it was changed to 65 years because of insufficient numbers of patients older than 70. There was no significant interaction between treatment and subgroup variable as defined according to the prespecified value for interaction (P < 0.01). The use of a cutoff of 40% rather than the prespecified 50% for the ejection fraction did not alter the results. There was no interaction for the presence or absence of ST-segment elevation, Q-wave loss, or R-wave loss. LAD denotes left anterior descending artery. *(Hochman et al.  NEJM Dec 7, 2006 p2405)*

Most Forest plots in trial reports look at several subgroup analyses, such as in Figure 10. This is for time to a composite primary outcome and hence hazard ratios (and their CIs) are displayed. One first concentrates on the overall estimate and the fact that its 95% CI overlaps one indicates no significant difference between PCI and medical therapy. Next, most subgroup CIs have substantial overlap with the overall point estimate at the top, which indicates a consistency of findings across subgroups.

The one exception is age. The two CIs for younger and older patients overlap only slightly, and the interaction test has P = 0.05. This might provoke some interest as an exploratory finding suggesting PCI may have more merit in older patients. However, the authors, aware of the dangers of false positive findings across multiple subgroup analyses, mention in the footnote that P < 0.01 was the tough pre-specified criterion for any claims of interaction. Figure 10 also tabulates four-year event rates by treatment and subgroup, which is a useful way of documenting absolute risk and how it varies by subgroup. For instance, event rates are higher for patients with ejection fraction < 50%. The figure's footnote is unduly long, and perhaps much of it should have been in the Methods section instead.

**Figure 11:**
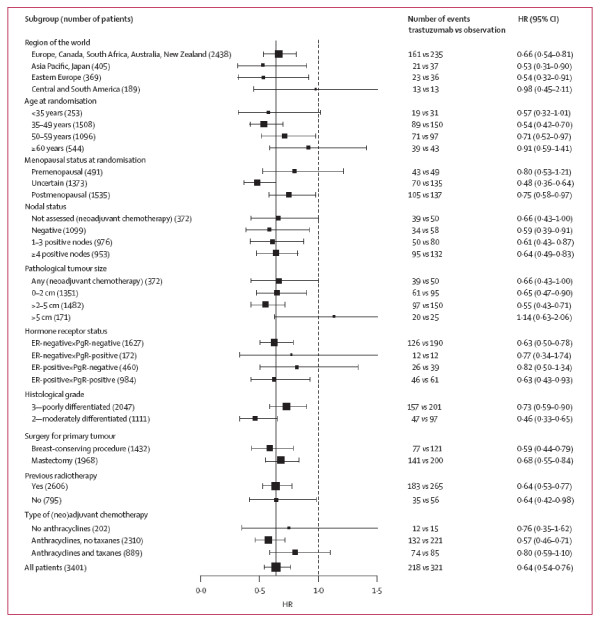
Exploratory disease-free survival subgroup analysis for 1 year of trastuzumab *vs *observation. *(Smith et al.  Lancet Jan 6, 2007 p33)*

Figure 11 clarifies some other features of a Forest plot. In all such plots the more events that occur in a subgroup the narrower the CI. To help the eye to focus on these more precise estimates, they are given a larger square blob, whereas in contrast small subgroups have a tinier square. Figure 11 also tabulates the numbers of patients with the event, by treatment and subgroup. This helps give reality to the plotted hazard ratios. The plot includes a vertical line at the overall effect, which helps the eye to spot any potentially deviant subgroups.

Figure 11 did not provide any interaction tests; instead the text includes the comment "there was no evidence of substantial heterogeneity...." The term HR is a little blunt; to state "hazard ratio" would be clearer. Again to give all HRs and CIs in both figure and tabular form is unnecessarily repetitious.

**Figure 12:**
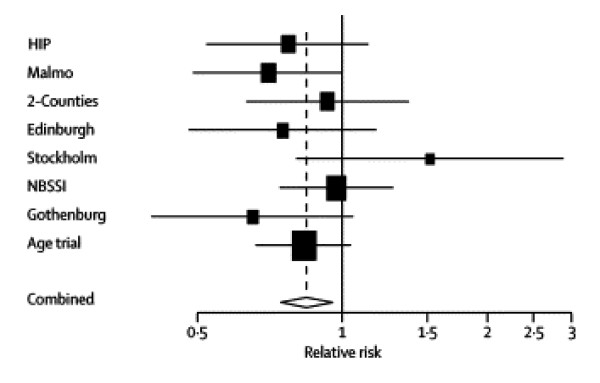
Breast cancer mortality results of the randomised mammography trials in women younger than 50 years. *(Moss et al.  Lancet Dec 9, 2006 p2059)*

The other main use of Forest plots is for meta-analyses, which display estimates from several related trials and combines them into an overall combined estimate. This is illustrated in Figure 12. To help the overall estimates and CI stand out it is usually shown as a diamond shape. Figure 12 is rather too minimalist as there is no additional data provided besides the plot itself. Also the plot does not identify which is the new trial. However, one can deduce that while no individual trial had a significant mortality reduction (assuming 95% CIs are plotted), the combined estimate's CI is narrower and wholly under one indicating overall significance. The smaller Stockholm trial is readily spotted as a potential outlier. Other occasional uses of Forest plot can be to plot treatment effects by different periods of follow-up, different outcome measures or different analysis methods. However, Forest plots are mostly for subgroup analyses of binary or time-to-event outcomes with odds ratio, relative risk or hazard ratio estimates.

**Figure 13:**
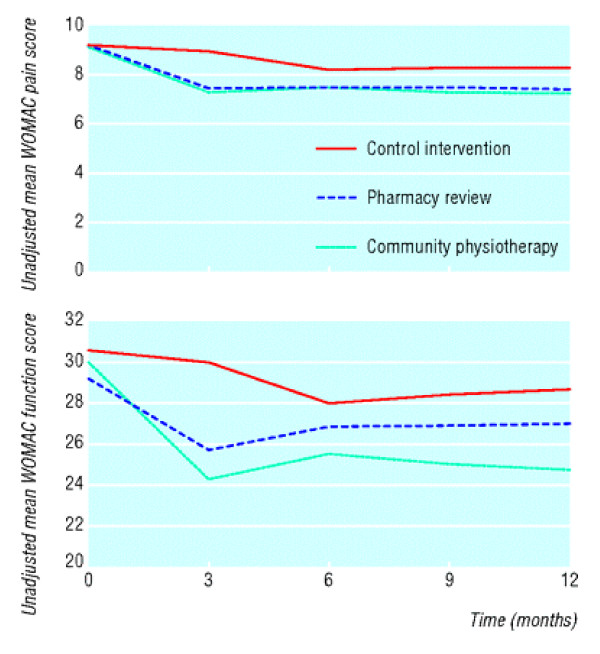
Mean Western Ontario and McMaster Universities osteoarthritis index (WOMAC) scores at recruitment and at 3, 6, and 12 months' follow-up. Top: WOMAC pain scores. Bottom: WOMAC function scores. *(Hay et al.  BMJ online Nov 11, 2006 p5)*

For trials with a quantitative outcome measure it is common to have repeated measures at fixed follow-up times, and usually also at baseline. It is then usual to plot the means by treatment over time. Figure 13 is an unduly simple example that lacks much important information. This only plots the means whereas it is good practice to also have standard error bars to illustrate the statistical uncertainty in each mean. It is also good practice to have symbols at each mean in addition to joined lines: this would make clear that measurements were made at 0, 3, 6 and 12 months but not at 9 months. From the figure alone one cannot determine how strong is the evidence for lower (better) scores in the pharmacy and physiotherapy groups. Also, there is an inconsistency of style in that the vertical axis of one plot starts at 0 while the other does not. Perhaps both plots should have shown the detail with a clearly indicated non-zero vertical origin. Lastly, there is no indication regarding numbers of patients, though admittedly such detail is in a separate Table.

**Figure 14:**
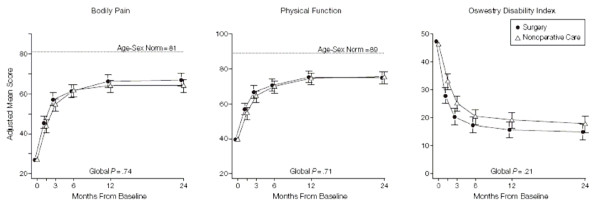
**Mean Scores Over Time for SF-36 Bodily Pain and Physical Function Scales and Oswestry Disability Index**. Age- and sex-weighted population normative scores are plotted for Medical Outcomes Study 36-Item Short-Form Health Survey (SF-36) scales. To enhance readability, the plot symbols and error bars for the surgical group are slightly offset. Error bars indicate 95% confidence intervals. *(Weinstein et al.  JAMA Nov 22, 2006 p2447)*

Figure 14 presents 95% CIs for each mean, slightly offset to enhance readability. We have a slight preference for standard error bars instead since they are roughly half the width. Furthermore error bars only need be shown in one direction, going up for the top line and down for the bottom line since their symmetry about each mean is known. Figure 14 usefully incorporates a global P-value, which clarifies that there is insufficient evidence of a treatment difference. Again, there is no indication of the numbers of patients involved.

**Figure 15:**
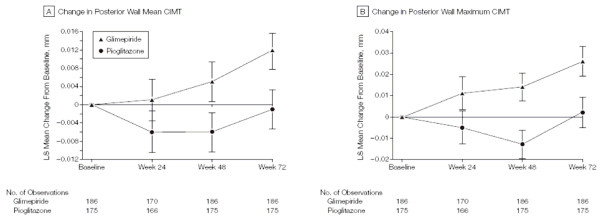
**Change From Baseline to Week 72 in Mean and Maximum CIMT of the Common Carotid Artery**. Values presented are least-square (LS) means using last observation carried forward. Error bars indicate SEs. A, Baseline LS mean, 0.771 (SE, 0.008) mm for pioglitazone and 0.779 (SE, 0.008) mm for glimepiride. Treatment-group difference (pioglitazone – glimepiride) at final visit, -0.013 (95% confidence interval, -0.024 to -0.002; *P *= .02). B, Baseline LS mean, 1.038 (SE, 0.0100) mm for pioglitazone and 1.042 (SE, 0.0100) mm for glimepiride. Treatment-group difference at final visit, -0.024 (95% confidence interval, -0.042 to -0.006; *P *= .008). CIMT indicates carotid intima-media thickness. *(Mazzone et al.  JAMA Dec 6, 2006 p2577)*

In Figure 15 the authors adopt a different (often better) approach by plotting mean changes from baseline, rather than means, with standard error bars. Analysis of covariance adjusting for baseline value is a preferred method of inference for such data [18], and this is what these authors mean by least-square (LS) means as explained in their Methods section. They have used last observation carried forward (LOCF) which is now regarded as less desirable than an appropriate repeated measures model assuming missing at random [19], but that is a separate issue from assessing the figure itself. The numbers of patients by group at each time are appropriately given below the x-axis, though it is puzzling as to why there are fewer at 24 weeks. The footnote to Figure 15 gives the primary inference regarding treatment differences at final visit, which is important detail that could alternatively have been in the main text.

**Figure 16:**
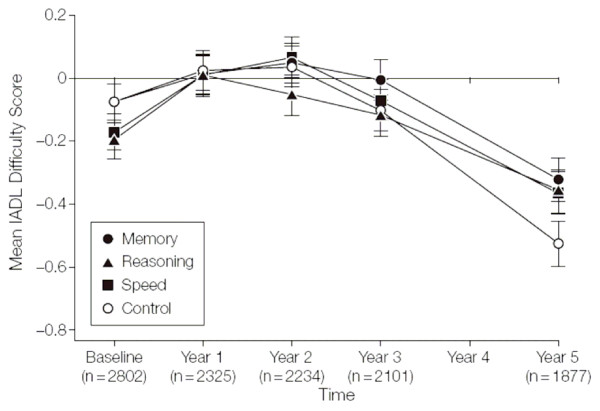
**Training Effects on Everyday Function by Self-reported Instrumental Activities of Daily Living (IADL) Difficulty Scores**. The mean scores are Blom-transformed. Error bars indicate SE. The sample sizes for each time point represent the number of cases with complete data for the IADL difficulty score. *(Willis et al.  JAMA Dec 20, 2006 p2811)*

Figure 16 illustrates the difficulty of plotting repeated measures with several treatment groups. Some of the points are hidden behind one another and the standard error bars are confusingly entangled. This could have been alleviated by having the four points offset slightly at each time. Also, with several treatments it may be better to not join the points with lines.

Since the main inference is about baseline adjusted mean changes this would have been conveyed better with a plot of mean changes rather than means. From the rest of the article, one deduces that all three intervention groups did somewhat better than the control group at five years, a fact hard to decipher from the figure. Note the 30% drop-outs by five years, which is usefully made clear in Figure 16.

Bar charts are occasionally used to display summary statistics such as means or percentages by treatment groups. However, many authors correctly decide that such relatively simple results are best shown in a table or text rather than as a figure.

**Figure 17:**
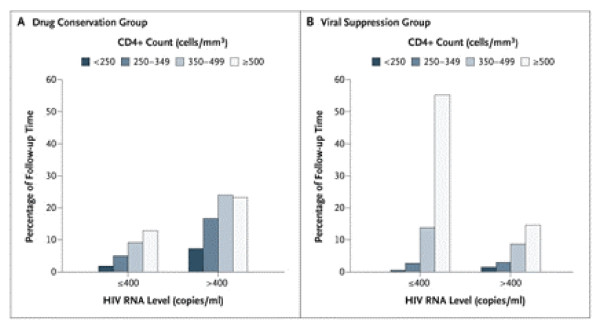
**Percentage of Follow-up Time during Which Participants Had a Specified CD4+ Count and HIV RNA Level**. For 28.8% of the 3666 person-years of follow-up in the drug conservation group (Panel A), the HIV RNA level was 400 copies per milliliter or less. Patients received antiretroviral therapy during 33% of the follow-up time. For 72.3% of the 3701 person-years of follow-up in the viral suppression group (Panel B), the HIV RNA level was 400 copies per milliliter or less. Patients received antiretroviral therapy during 94% of the follow-up time. *(SMART Study Group.  NEJM Nov 30, 2006 p2288)*

Figure 17 illustrates why a bar chart figure can sometimes be of descriptive value, since the joint patterns of CD4 count and HIV RNA level during follow-up are "brought to life" more readily than could be achieved in a table.

**Figure 18:**
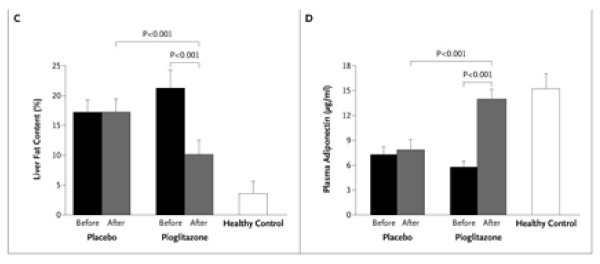
**Hepatic Fat Content Assessed by Means of Magnetic Resonance Spectroscopy before and after the Study Treatment (Panel C); and Plasma Adiponectin Concentrations before and after the Study Treatment (Panel D)**. In Panels C and D, P < 0.001 for the comparisons of the pioglitazone and placebo groups with the healthy controls both at baseline and at 6 months, except that the comparison of post-treatment plasma adiponectin concentrations in the pioglitazone group and the healthy controls was not significant. I bars and T bars denote standard deviations.*(Belfort et al.  NEJM Nov 30, 2006 p2302)*

Figure 18 illustrates use of a bar chart for displaying means of a quantitative outcome by treatment group, before and after treatment. However, such means might be better plotted as points (as in Figures 13, 14, 15 and 16) rather than as bars[20]. Statistical uncertainty is usefully presented as error bars: the footnote states they are standard deviations, but we suspect they intend standard errors which would be more appropriate since it is the precision of each estimated mean rather than the individual variation that matters here. Figure 18 does not inform us as to the number of patients for each mean.

**Figure 19:**
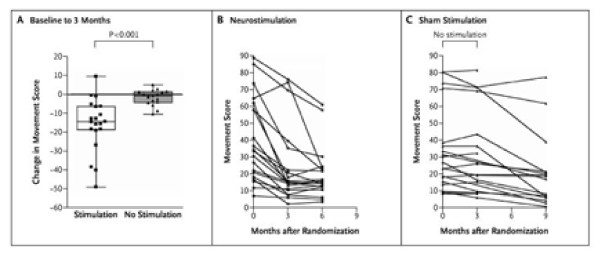
**Changes in Movement Scores from Baseline to 3 Months and the Effects of 6 Months of Neurostimulation, as Compared with Sham Stimulation**. As shown in Panel A, patients receiving effective high-frequency neurostimulation of the internal globus pallidus for 3 months had a significantly greater improvement in dystonic symptoms, as assessed by blinded ratings with the use of the Burke-Fahn-Marsden Dystonia Rating Scale, than did patients receiving sham stimulation. Each symbol denotes the change in scores from baseline to 3 months. The box plots represent the median and interquartile range. I bars show the range for each group. The changes in movement symptoms throughout the trial are shown for patients who were initially assigned either to the neurostimulation group (Panel B) or the sham-stimulation group (Panel C). *(Kupsch et al.  NEJM Nov 9, 2006 p1983)*

There are few instances where individual patient data are displayed in a trial report. This is best confined to relatively small trials, since such plots become too cluttered with large numbers of patients. Figure 19 is one useful plot of such individual data, which helps one to visualise the individual falls in movement score in the neurostimulation group. The accompanying box plot clarifies further, with the median and interquartile ranges in the two groups being clearly separated.

**Figure 20:**
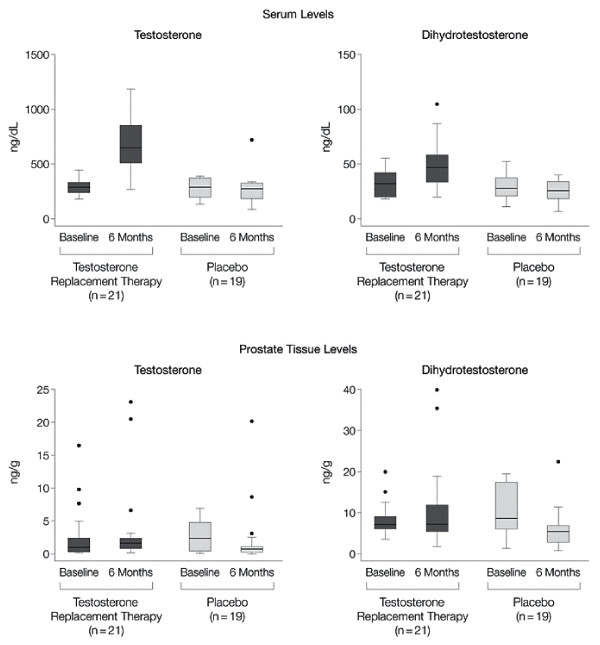
**Effects of Treatment on Serum and Prostatic Androgen Levels**. Both testosterone and dihydrotestosterone levels increased in serum after 6 months of treatment with testosterone replacement therapy (*P *< .001 by signed rank test). However, despite an increase in serum levels for testosterone to the mid-normal range, prostate tissue levels of the androgens did not change significantly. Boxes contain 50% of data with the inside horizontal line representing the median value; whiskers contain 100% of data, except for statistical outliers shown as individual data points. *(Marks et al.  JAMA Nov 15, 2006 p2356)*

Figure 20 is another example of a box plot. The footnote states "whiskers contain 100% of data, except for statistical outliers shown as individual points", though what constitutes an outlier in undefined, and possibly unnecessary. They are perhaps best called extreme values since the term "outlier" incorrectly implies they are invalid readings. With such skew distributions these plots would have been clearer on a log scale.

## Discussion

Figures are a key element of any trial report. They are often more likely to be noticed by readers than text or tables, and to be disseminated in conferences and discussions, since by their very nature figures catch the eye more readily, and hence have the potential to convey key results more fully and immediately. Figures can reveal unexpected (and expected) patterns in data and graphs of model estimates can encapsulate the entire picture of what was learnt in a study, much more than can be done in a table. To date, little attention has been paid to what constitutes good practice for producing figures in trial reports. Each journal has its own approach to use of figures, but the key choice rests with what figures authors include in their submitted articles.

Our survey of three months of trial reports in five key journals illustrates on the one hand that the great majority of figures are of four main types (Flow diagram, Kaplan Meier plot, Forest plot and Repeated measures over time), but on the other hand there is a great diversity of style in the way those Figures are presented.

The display of statistical uncertainty, i.e. standard errors (SEs) or CIs, is an important component of many figures. When comparing two groups it is useful for readers to have insight into how the extent of overlap between SEs or between CIs is related to the strength of evidence for a difference between groups [21]. The following rough guide works well when the two groups have SEs of similar magnitude:

1) If there is any overlap between the standard error bars then the difference is not statistically significant

2) If there is a gap between the standard error bars and that gap itself exceeds one standard error then the difference is significant, at P < 0.035 in fact. Thus, a lesser gap may fall short of conventional significance

3) If the 95% CIs do not overlap then we have strong evidence of a difference, P < .006 in fact. So, a slight overlap between two 95% CIs may still be statistically significant.

Of course, this guide should not substitute for the formal presentation of P-values for comparisons of key interest.

A cynic might observe that i) authors lack imagination and are over-conservative in their use of figures and ii) authors are sloppy in the way they actually present figures. The first point may be unduly harsh, since clinical trials have a limited number of data types, and over time it has become evident which types of figure work in practice. Also, unconventional uses of figures, while having creative potential may carry the risk that some readers struggle to understand and interpret them. Nevertheless some types of figure may at present be underutilized, for instance appropriate displays of individual patient data.

We feel there is more justification in the second criticism above as regards sloppiness and inconsistencies in style. Accordingly, we devote the rest of this Discussion to a list of Recommendations for future practice.

## Recommendations

First some issues relating to **figures in general**:

1)One needs to decide which results merit a figure rather than a table. Some figures (e.g. Kaplan Meier plots) would be cumbersome as a Table while others (e.g. a bar chart of percentages) may be better in tabular form or in the text.

2) Every figure needs the following: a good legend, clear labelling, clarity of presentation and to stand alone in its comprehensibility rather than needing explanation in the text.

3) Figures should clearly identify each treatment group, and require care in use of colours and background shading since many readers use black-and-white copies.

4) Figures should indicate the numbers of patients by treatment group in each analysis presented.

5) Figures should display appropriate measures of uncertainty, e.g. standard error bars or CIs.

6) Figures should often state the primary inferences to be derived from them, e.g. estimates of treatment effect, their CIs and P-values, since visual inspection alone could lead to misleading interpretations by the reader.

7)The creation of high-quality figures requires careful attention to overall principles of graph construction and visual display as developed by specialists in this field [1,2,4,6,7].

The following recommendations relate to the four main types of Figure:

### A) Flow Diagram

8) Every trial report should include a flow diagram, in line with CONSORT guidelines [11,12].

9) The flow diagram should include the numbered flow of participants throughout the trial, including the numbers screened and eligible prior to randomization.

10) It is particularly important to provide the numbers in each group lost to follow-up or excluded from analysis for other reasons.

11) Some flow diagrams can become indigestible with too many repeat words, especially with several treatment arms. These may be better displayed as a Table without loss of information.

### B) Kaplan Meier plot

12) Plots should include numbers at risk over time under the time axis.

13) The plot should not extend too far in time, to avoid the numbers at risk becoming unduly small.

14) Plots with relatively low event rates should be displayed going up (i.e. cumulative percent with event on the vertical axis) so that the detail is discernable.

15) Plots should often include standard error bars at appropriate time points to convey statistical uncertainty. To date this is rarely done.

### C) Forest plot

16) In addition to estimates and 95% CIs for various subgroups, Forest plots should also include the overall estimate and its CI. Drawing a vertical dotted line at the overall estimate helps readers to spot any consistency (or otherwise) across subgroups.

17) One can usefully use varying sizes of square at each estimate to indicate which subgroups are based on a lot (or a little) data.

18) For plots of hazard ratio, odds ratio or relative risk a log scale is often preferable, leading to symmetric CIs.

19) The risk scale should provide an appropriate degree of detail, and make clear which direction indicates which treatment is better.

20) Forest plots can usefully tabulate for each subgroup some of the following: the numbers of patients and numbers with events by treatment, the estimate and its CI and the interaction test P-value. However, this should not result in excessively detailed information for what is an exploratory subgroup analysis.

21) Interaction tests should be reported rather than subgroup P-values. That is, the difference between "significant" and "non-significant" subgroups may not be statistically significant[22].

### D) Repeated measures plot

22) The points for each estimate (usually means) at each time point should be clearly marked and joined by lines for each treatment in a clearly identified manner. With several treatment groups it may be clearer to identify groups by symbols rather than by lines.

23) Measures of uncertainty, usually standard errors bars, should be attached to each point, and the number of patients included should be under the time axis.

24) It is useful to slightly stagger the groups so means and standard errors don't overlap confusingly.

25) It is often better to plot mean changes from baseline, rather than means, using analysis of covariance to present baseline adjusted mean changes.

26) The method of analysis used to make inferences from the repeated measures should be briefly stated on the plot, and it may be useful to add some overall estimate of treatment effect with CI and P-value.

## Conclusion

In conclusion, we hope these useful pointers enhance the quality of clinical trial reports with respect to use of figures. A similar enquiry to this may be of value for other type of study eg reports of observational studies in epidemiology, so that all journal articles pay appropriate attention to the informative use of figures.
